# Anti-CRISPR-Based and CRISPR-Based Genome Editing of *Sulfolobus islandicus* Rod-Shaped Virus 2

**DOI:** 10.3390/v10120695

**Published:** 2018-12-08

**Authors:** David Mayo-Muñoz, Fei He, Jacob Bruun Jørgensen, Poul Kári Madsen, Yuvaraj Bhoobalan-Chitty, Xu Peng

**Affiliations:** Danish Archaea Centre, Department of Biology, University of Copenhagen, Ole Maaløes Vej 5, 2200 Copenhagen, Denmark; damamuo@gmail.com (D.M.-M.); feihe2010@163.com (F.H.); csp411@alumni.ku.dk (J.B.J.); xps228@alumni.ku.dk (P.K.M.); yuvarajb12@gmail.com (Y.B.-C.)

**Keywords:** anti-CRISPR-based genome editing, selection marker, CRISPR-based genome editing, core genome, accessory genes, essentiality, virus-host interaction

## Abstract

Genetic engineering of viruses has generally been challenging. This is also true for archaeal rod-shaped viruses, which carry linear double-stranded DNA genomes with hairpin ends. In this paper, we describe two different genome editing approaches to mutate the *Sulfolobus islandicus* rod-shaped virus 2 (SIRV2) using the archaeon *Sulfolobus islandicus* LAL14/1 and its derivatives as hosts. The anti-CRISPR (Acr) gene *acrID1*, which inhibits CRISPR-Cas subtype I-D immunity, was first used as a selection marker to knock out genes from SIRV2M, an *acrID1*-null mutant of SIRV2. Moreover, we harnessed the endogenous CRISPR-Cas systems of the host to knock out the accessory genes consecutively, which resulted in a genome comprised solely of core genes of the 11 SIRV members. Furthermore, infection of this series of knockout mutants in the CRISPR-null host of LAL14/1 (Δarrays) confirmed the non-essentiality of the deleted genes and all except the last deletion mutant propagated as efficiently as the WT SIRV2. This suggested that the last gene deleted, SIRV2 *gp37*, is important for the efficient viral propagation. The generated viral mutants will be useful for future functional studies including searching for new Acrs and the approaches described in this case are applicable to other viruses.

## 1. Introduction

Viruses are recognized as the most abundant biological entities on Earth. There are an estimated 10^31^ viruses on the planet [1] which outnumber their hosts by approximately tenfold in most environments [2,3]. Metagenomics has shown the great diversity of viral communities. There are possibly 500 different viral genotypes in 200 L of seawater and around one million different viral genotypes in 1 kg of marine sediment [4]. However, viruses are still poorly understood with most of their genes having an unknown function and showing little or no sequence similarity to genes in databases [5]. Even though structural, transcriptomic, and proteomic studies have shed some insight into the molecular mechanisms of the virus life cycle, efficient genetic manipulation is of great importance to facilitate the functional characterization of many of these viral genes.

In general, genetic engineering of viruses is challenging. While non-lytic viruses can be manipulated relatively easily by the introduction of a selection marker so that the host carrying the recombinant viral genome survives the selection, for lytic viruses, only markers that are essential for their propagation can be used for the genetic manipulation. For example, *Escherichia coli trxA* and *cmk* genes are required for T7 phage propagation but not for host growth and they both can be used as markers for T7 phage engineering [6]. Only T7 phages that acquired the marker gene can propagate in a host lacking the respective marker gene. However, these markers are scarce and only identified in a few well-studied phages. To circumvent this, the Bacteriophage Recombineering of Electroporated DNA (BRED) strategy was employed for the manipulation of lytic mycobateriophage [7,8]. In this case, BRED phage DNA and a targeting template (either ssDNA or dsDNA with homologous arms flanking the mutation sites of phage) are co-electroporated into a host. Furthermore, phage-derived recombinases are exploited to induce high levels of homologous recombination between the phage genome and the targeting template. However, this system is hard to use in strains refractory to electroporation. For archaeal viruses, genetic engineering becomes even more crucial since they exhibit great genetic diversity and only an extremely low percentage of their genes have detectable homologs in public databases [9,10]. In previous studies, tedious methods such as long inverse PCR (LIPCR) [11,12,13,14], transposon mutagenesis [11,14], or infectious clones [15] have been used for the construction of viral mutants in *Sulfolobus* spindle-shaped virus (SSV) and *Sulfolobus* turreted icosahedral virus (STIV). Both of these viruses have circular dsDNA genomes.

More recently, Clustered Regularly Interspaced Short Palindromic Repeats (CRISPR) and their associated genes (*cas*) systems have come into play. CRISPR-Cas constitutes a widely-spread adaptive immune system in bacteria and archaea [16,17]. CRISPR loci consist of several *cas* genes and arrays of short repeats separated by variable sequences originated from mobile genetic elements especially viruses and plasmids. crRNAs expressed from the CRISPR arrays form effector complexes together with Cas proteins, which guide them for invader silencing [18,19]. CRISPR-Cas systems especially type II (Cas9) have been harnessed for genetic engineering of many organisms such as bacteria, yeast, plants, and animals. In the first attempt, *E. coli* subtype I-E CRISPR-Cas system was used to generate a deletion of two genes from T7 phage genome [20]. In addition, type II CRISPR-Cas system has also been exploited for genetic manipulation of viruses. In one case, CRISPR-Cas9 was reported to be a valuable tool for non-integrating adenoviral gene deletion and exchange [21]. Subtype II-A CRISPR-Cas system from *Streptococcus thermophiles* was also used for point mutation, deletion, and gene swap in *Streptococcus* phage 2972 [22].

In this scenario, we report on two different approaches for editing the genome of an archaeal virus known as the *Sulfolobus islandicus* rod-shaped virus 2 (SIRV2), which carries a linear dsDNA of 35,498 bp with covalently-closed ends and encodes 54 genes [23,24,25]. The first approach involves an anti-CRISPR (Acr) protein as a selection marker while the second approach was marker-less by harnessing the host endogenous CRISPR-Cas for editing. Acrs have been discovered in a number of viruses to date from several narrow groups of bacteria and archaea such as *Pseudomonas*, *Listeria*, *Streptococcus*, *Moraxella*, and *Sulfolobus*. Forty different families of Acr proteins have been found against CRISPR-Cas subtypes I-C, I-D, I-E, I-F, II-A, II-C, and V-A [26,27,28,29,30,31,32,33,34,35,36], mostly by using a guilt-by-association bioinformatics approach based on anti-CRISPR-associated genes [30]. AcrID1 (SIRV2 *gp03* and SIRV3 *gp02*) is the first example of Acr found in the archaea domain and the first against CRISPR-Cas type I-D system [29]. In this work, *acrID1* was exploited as a selection marker to delete non-essential or essential genes in the viral genome.

This research paper consists of two major parts. The first part involves gene knockout from SIRV2M, an *acrID1*-null mutant of SIRV2 [29], using *acrID1* (SIRV3 *gp02*) as a selection marker in the *Sulfolobus* host carrying CRISPR-Cas type I-D immunity. In the second part, we knocked out consecutively the accessary genes from another mutant of SIRV2 using a CRISPR-based genome editing method and obtained a knockout mutant that carries only the core genome of the 11 SIRVs known as SIRV1–SIRV11 [37]. The infectivity of the different viral mutants was also studied.

## 2. Material and Methods

### 2.1. Strains and Growth Conditions

*Sulfolobus islandicus* LAL14/1 and its derivatives carrying *pyrF* deletion (Δ*pyrF*) [38] and all five CRISPR arrays deletion (Δarrays) [38] were used for infection and transformation. *E. coli* DH5α was used for cloning of the plasmids. 

*Sulfolobus* cells were grown aerobically at 78 °C and 150 rpm in a GCVY medium, which contains basic salts medium [39] supplemented with 0.2% glucose, 0.2% casamino acids, 0.005% yeast extract, and a vitamin mixture or SCV medium containing basic salts medium supplemented with 0.2% sucrose, 0.2% casamino acids, and a vitamin mixture. Uracil (20 μg/mL) was added to the medium for the cultivation of the Δ*pyrF* strains. Transformed *E. coli* cells were grown overnight at 37 °C in LB (Luria–Bertani broth) agar supplemented with 100 μg/mL ampicillin. Single colonies were inoculated into 20 mL of LB media supplemented with 100 μg/mL ampicillin and grown overnight at 37 °C and 200 rpm.

### 2.2. Cloning

All gene fragments were amplified by using PCR or fusion PCR with a Phusion Hot Start II DNA Polymerase (2 U/µL) (F549L, Thermo Scientific™, Waltham, MA, USA) and primers listed in Table S1. All primers and spacers were ordered as ssDNA (Integrated DNA Technologies, Inc., Coralville, IA, USA) and are listed in Table S1. PCR products were analyzed by using agarose gel electrophoresis. PCR or digested products were purified using the GeneJET PCR Purification Kit (K0702, Thermo Scientific™). Digested fragments and vectors were ligated using the T4 DNA ligase kit (5 U/µL) (EL0011, Thermo Scientific™), according to the manufacturer’s instructions. The ligated DNAs were transformed into *E. coli* DH5α, analyzed by colony PCR using Taq DNA Polymerase and plasmid clones, which were purified using the Plasmid DNA Mini Kit I (D6943-02, Omega, Norcross, USA). The inserts of the resultant clones were analyzed by using PCR, which was followed by DNA sequencing (Eurofins Genomics, Ebersberg, Germany).

### 2.3. Construction of Anti-CRISPR-Based Genome Editing Plasmids

First, we constructed the pJET1.2 vectors with homologous flanking arms. Second, the *acrID1* marker was digested with *Nhe*I and *Kpn*I restriction enzymes (ER0971, ER0521, Thermo Scientific™) and inserted between the homologous flanking arms in pJET1.2, which yielded pJET1.2 plasmids listed in Table S1. SIRV2 *gp49* was cloned into pEXA2 [40] for its expression.

### 2.4. Construction of CRISPR-Based Genome Editing Plasmids

CRISPR-based genome-editing plasmids (Table S1) were constructed individually by cloning a mini-CRISPR array carrying a single spacer and a deletion mutant allele of the target gene into the basic genome-editing plasmid pGE1 [38]. pGE1 contains *LguI* restriction sites that were oppositely oriented and flanked by two CRISPR type I-A repeats as well as *PaeI*-*XhoI* restriction sites for the insertion of homologous recombination sequences. crRNA generated from the mini-CRISPR array can be used by type III effector complexes [41]. Additionally, for selective purposes, it contains a uracil synthesizing gene [42].

First, a protospacer is identified in the gene of interest, which will be targeted by the CRISPR-Cas immune system once the corresponding crRNA is provided. It is important to consider that type III systems function only when the crRNA is complementary to a transcript. Under these circumstances, a protospacer—39 nt long—on the template strand of the target gene was selected. Afterwards, a spacer was constructed based on the selected protospacer of the target gene by mixing two complementary oligonucleotides (Table S1), which was followed by heating at 95 °C for 10 min and subsequently cooling down gradually to room temperature. The resulting dsDNA carrying 3 nt protruding ends—AAC and AGC—was ligated into the *LguI* site of the linearized pGE1 (FD1934, Thermo Scientific™), which yielded plasmids carrying a mini-CRISPR array.

Respective donor DNA fragments containing the expected deletion mutant allele of each target gene were obtained by using overlap extension PCR [43] from a SIRV2M_II_ (Table 1) template. The primers were designed for PCR amplification of two homologous DNA sequences (left and right arms) flanking the target gene from the viral genome and, crucially, did not include the protospacer. After fusion of both arms, the PCR products were digested with *PaeI* and *XhoI* restriction enzymes (FD0604, FD0694, Thermo Scientific™) and purified again. In the case of target genes that are adjacent in the genome—e.g., *gp10*, *gp11*, and *gp12*— a donor DNA was designed for the deletion of all of them at a time. It is fundamental to consider maintaining intact promoters and terminators of neighboring genes that might be within the target gene sequence. The resulting restriction DNA fragments were inserted into their cognate mini-CRISPR array plasmids at the same site, which gave rise to pGE1 constructs listed in Table S1.

### 2.5. Electroporation Procedure

Electroporation was used to transform *S. islandicus* with each pJET1.2-based or pGE1-based construct, according to a procedure described previously [44], with the following modifications: (1) all manipulations were carried out at room temperature, (2) after electroporation of 500 ng DNA into 50 μL *Sulfolobus* competent cells (OD_600_ around 10), the cells were immediately transferred into 950 μL pre-warmed basic salts medium (pre-mixed with SIRV2M, MOI of 1, only in the case of pJET1.2 transformation) and incubated at 75 °C for 30 min without shaking, and (3) 100 μL of cells were mixed with 5 mL of 2x SCV and 5 mL 0.4% Gelrite^®^and plated onto pre-warmed 1.4% Gelrite^®^ (71010-52-1; Roth) plates containing 2x SCV medium. After the top layer was set, the plates were put into a tightly closed plastic box, and incubated at 78 °C for five to seven days.

### 2.6. Construction of Knockout Mutants and Knockout Screening by PCR

In the case of the CRISPR-based genome editing, the cells transformed with knockout plasmids were grown in SCV medium (containing no uracil for the selection of the plasmids) and were infected with the corresponding virus for genome editing. The cultures were grown from OD_600_ 0.1 for 1 h and infected with 10 µL of the previous deletion mutant for the next gene knockout. After 48 h of infection, viruses were harvested by centrifuging at 10,000× *g* for 6 min and the supernatant was diluted 10^6^ times into fresh Δarrays cells carrying the corresponding genome editing plasmid. The process was repeated 4 to 5 times for complete removal of the WT virus.

All deletion mutants were screened by PCR amplification of the WT target gene and its mutated allele by using primers listed in Table S1. The resulting PCR products were analyzed by agarose gel electrophoresis and by DNA sequencing (Eurofins Genomics, Ebersberg, Germany).

### 2.7. Plaque Assay

Three mL of LAL14/1 Δarrays cells (OD_600_ around 0.2) were premixed with serial dilutions of the corresponding virus and incubated at 78 °C and 150 rpm for 30 min. The infected cultures were then mixed with 3 mL of 0.4% Gelzan™ CM (Gelrite^®^, G1910, Merck KGaA, Darmstadt, Germany) and plated onto pre-warmed 1.4% Gelzan plates containing 2x SCV medium. After the top layer was set, the plates were put into a plastic box, tightly closed, and incubated at 78 °C for two days. The observed circular zones of clearing (plaques) were due to viral replication and lysis of the *Sulfolobus* host. The plaque forming units (PFU) were calculated from the plates containing a quantifiable number of plaques.

## 3. Results and Discussion

### 3.1. Knockout of a Non-Essential Gene (SIRV2 Gp29) and an Essential Gene (SIRV2 Gp49) from SIRV2M Genome Using AcrID1 as a Selection Marker

We reported recently that *acrID1* flanked with recombination arms can be inserted into the SIRV2M genome to evade the CRISPR-Cas I-D immunity [29]. In accordance, the first part of this work was to test whether *acrID1* could be used as a selection marker to knock out genes from the SIRV2M genome. To this end, we randomly selected a non-essential gene (SIRV2 *gp29*) and an essential gene (SIRV2 *gp49*) for the knockout [44,45].

**Figure 1:**
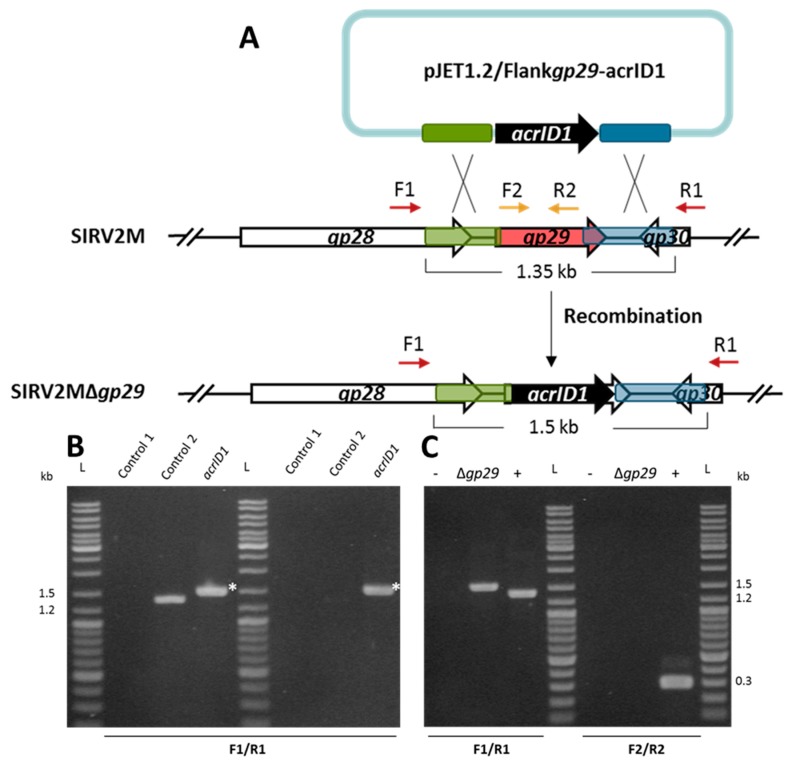
Knockout of the non-essential gene SIRV2 *gp29* by using anti-CRISPR-based genome editing. (**A**) Schematic illustration of the recombination (double crossover) between SIRV2M and *acrID1* fragment (black arrow) cloned between the homologous arms (green and blue boxes) in pJET1.2. Genes are depicted by arrows. F1/R1 primers, which are indicated by dark red arrows above the genome map, amplify a region covering the deletion target while F2/R2, which is depicted in gold, amplify a region within the deletion target (red arrow). (**B**) PCR analysis of the recombination events as depicted in (**A**) where the size of PCR fragments is also indicated. Supernatants of the cultures containing the plasmid (*acrID1)* two days post electroporation (left panel) and two days post dilution (1000 times into fresh LAL14/1 Δ*pyrF* cells) (right panel) were used as a DNA template in the PCR reactions. Control 1, water was electroporated into the cells but no SIRV2M was added. In control 2, water was electroporated into the cells after the SIRV2M was added. The PCR bands derived from recombinant viruses are labeled with white asterisks. (**C**) PCR verification of gene deletion in SIRV2MΔ*gp29* after two more times dilution (1000 times into fresh LAL14/1 Δ*pyrF* cells). PCR was conducted with “Δ*gp29* check primers” (Table S1) using supernatant from the culture carrying SIRV2MΔ*gp29* (Δ*gp29*). +, positive control using the SIRV2M virion as the PCR template, − as a negative control in “control 2” in (**B**), L as a DNA size ladder.

The SIRV2 *gp29*, which is an ORF encoding 156 amino acids, is not part of the core genome of the 11 SIRV members known as SIRV1-SIRV11 (PMID:28534836) [37] and, thus, is likely to be non-essential for viral propagation. As depicted in Figure 1A, a PCR product containing *acrID1* flanked by the upstream (495 bp) and downstream (441 bp) sequences of *gp29* was cloned into the bacterial plasmid pJET1.2, which does not replicate in *S. islandicus*. The plasmid was electroporated into LAL14/1 Δ*pyrF*, which carries all the wild type CRISPR-Cas systems including the I-D immunity [38]. SIRV2M was added immediately, which was followed by incubation at 78 °C for two days. Potential knockout of *gp29* through recombination between the homologous sequences of the plasmid and the SIRV2M genome (Figure 1A) was monitored by PCR amplification of the SIRV2 *gp29* region using virion DNA in culture supernatant as a template, which revealed a substitution of *gp29* with *acrID1* (Figure 1B, left panel). The culture supernatants were then diluted 1000 times into fresh LAL14/1 Δ*pyrF* cells and PCR was performed again after two days of incubation (Figure 1B, right panel). This indicated that the recombinant virus quickly became predominant and was maintained at a stable condition. To obtain the pure *gp29* deletion mutant (Δ*gp29*), the culture supernatant was then diluted 1000 times into fresh LAL14/1 Δ*pyrF* cells for two more rounds and the viral purity was checked by PCR amplification. No band was detected with the primers located inside the *gp29* gene indicating the high purity of the mutant virus (Figure 1C). Thus, we succeeded to exploit *acrID1* as a selection marker for the knockout of an inessential gene.

**Figure 2:**
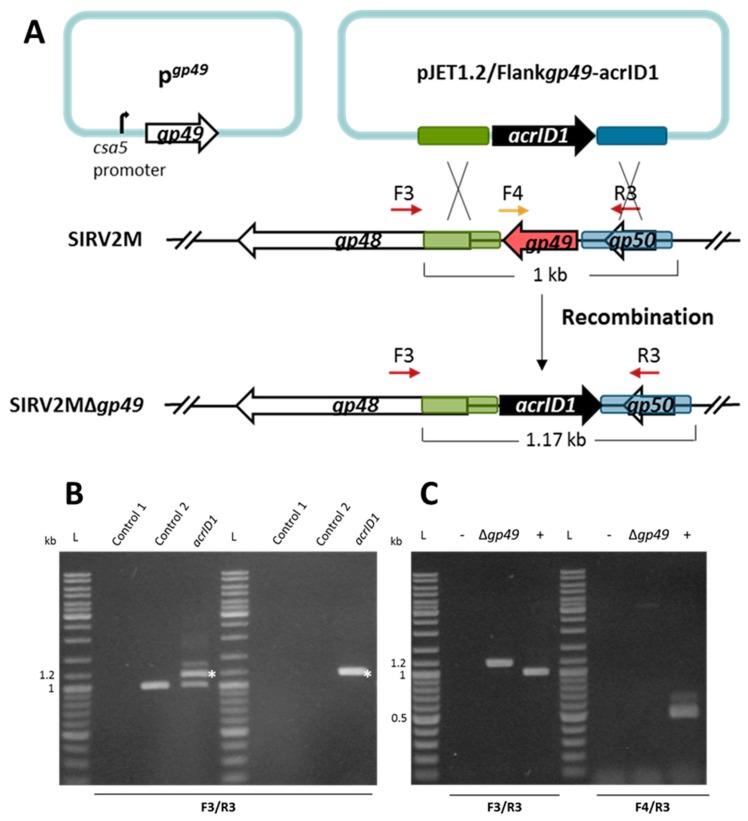
Knockout of the essential gene SIRV2 *gp49* by using the anti-CRISPR-based genome editing. (**A**) Right panel: schematic illustration of the recombination (double crossover) between SIRV2M and *acrID1* fragment (black arrow) cloned between the homologous arms (green and blue boxes) in pJET1.2. Genes are depicted by arrows. F3/R3 primers, which are indicated by dark red arrows above the genome map, amplify a region covering the deletion target while F4 (depicted in gold)/R3 amplify a region from the deletion target (red arrow). The *gp49*-containing plasmid (p*^gp49^*, left panel) was transformed into the cells prior to the knockout experiment. (**B**) PCR analysis of the recombination events as depicted in (**A**) where the size of the PCR fragments is also indicated. Supernatants of the cultures two days post electroporation with the *acrID1*-containing pJET1.2 plasmid (left panel) and two days post dilution (1000 times into fresh LAL14/1 Δ*pyrF*/p*^gp49^* cells) (right panel) were used as the DNA template in the PCR reactions (*acrID1*). Control 1: water was electroporated into the cells but no SIRV2M was added. Control 2: water was electroporated into the cells after which SIRV2M was added. The PCR bands derived from recombinant viruses are labeled with white asterisks. (**C**) PCR verification of gene deletion in SIRV2MΔ*gp49* after two more times dilution (1000 times into fresh LAL14/1 Δ*pyrF*/p*^gp49^* cells). PCR was conducted with “Δ*gp49* check primers” (Table S1) using supernatant from SIRV2MΔ*gp49*-containing cells (Δ*gp49*). (+) positive control using SIRV2M virion as the PCR template; −, negative control as “control 2” in (**B**) L, DNA size ladder.

Next, we attempted to knock out an essential gene. SIRV2 *gp49* (ORF98) encodes the only protein component of virus-associated pyramids (VAPs), which are assembled on the host cell surface and open up at the final stage of infection to facilitate the release of virus particles [46,47]. Accordingly, the VAP protein *gp49* is considered to be essential for the lytic virus life cycle. In order to knock out this gene, we introduced a plasmid-borne *gp49* into LAL14/1 Δ*pyrF* where *gp49* was placed under the control of a highly strong promoter known as the *csa5* promoter from the host [48]. This ensures the availability of enough VAP protein before the knockout takes place. The *gp49*-containing LAL14/1 Δ*pyrF* cells (LAL14/1 Δ*pyrF*/p*^gp49^*) were then used to knock out *gp49* from SIRV2M using the same strategy that was applied to the previously mentioned knockout of *gp29* (Figure 2A). As shown in Figure 2B, the recombination between the *acrID1*-containing plasmid and SIRV2M genome was already detectable two days post electroporation of the plasmid and the addition of SIRV2M in the culture (left panel). Moreover, as with the gp29 knockout, the recombinant virus remained stable after a 1000 times dilution into fresh cultures (right panel). PCR involving one primer derived from the *gp49* sequence demonstrated that a pure *gp49* knockout viral strain (Δ*gp49*) was obtained after two more rounds of a 1000 fold dilution of the virus into fresh cultures (Figure 2C).

**Figure 3:**
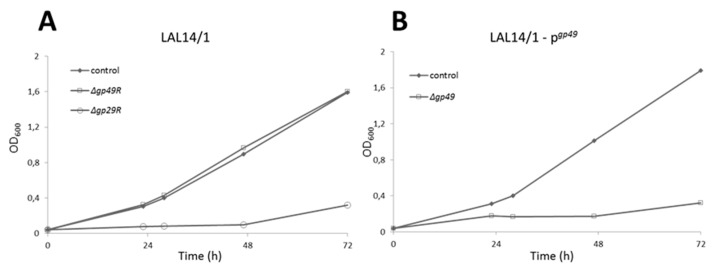
Growth curves showing the non-essentiality of *gp29* and the essentiality of *gp49*. (**A**) LAL14/1: ∆*pyrF* cells were transformed with an empty vector. (**B**) LAL14/1—p*^gp49^*: ∆*pyrF* cells transformed with p*^gp49^***.** Virus was added at time 0. No virus was added in the control culture.

The infectivity of SIRV2MΔ*gp29* and SIRV2MΔ*gp49* were then tested in LAL14/1 Δ*pyrF*, which carries the wild type CRISPR-Cas systems. Due to the insertion of *acrID1*, SIRV2MΔ*gp29* gained the infectivity in LAL14/1 (Figure 3A), which also demonstrated the inessentiality of *gp29*. However, *gp49* appeared to be essential for the lytic life cycle of the virus since SIRV2MΔ*gp49* is not able to cause growth retardation of the host cells (Figure 3A) unless a plasmid-borne *gp49* was provided (Figure 3B).

### 3.2. CRISPR-Based Viral Genome Editing Approach

Even though the previously mentioned knockout approach is efficient, it leaves the selection marker *acrID1* in the recombinant viral genome, which makes it difficult for consecutive deletions of other genes. Previously, CRISPR-Cas systems were harnessed for genome editing in the model archaeon *Sulfolobus* [41], but, so far, no CRISPR-based genome editing approach has been applied to archaeal viruses. Recently, SIRV2 *gp48*, which is a non-core gene of the 11 SIRVs, was deleted from SIRV2M for a comprehensive functional study (Bhoobalan-Chitty et al., in preparation) from which we initiated the CRISPR-based consecutive gene deletions.

**Figure 4:**
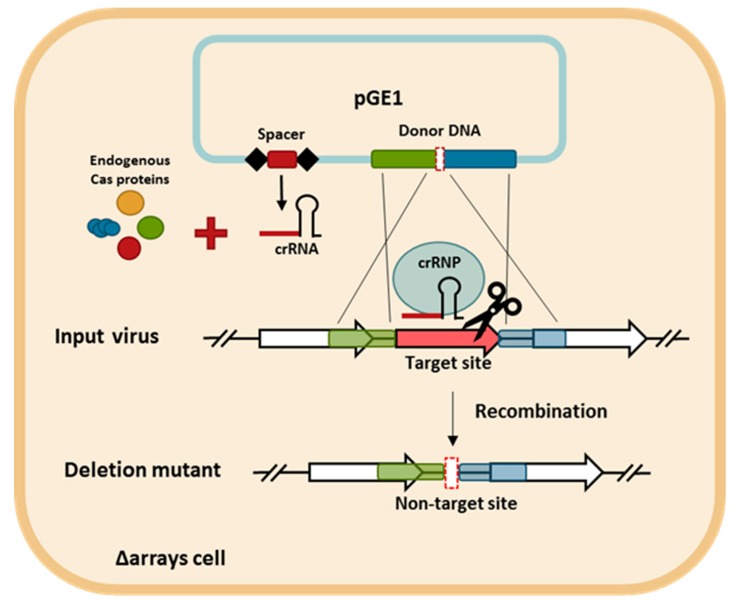
The schematic diagram of the CRISPR-based genome editing approach. The spacer and the donor DNA are cloned into pGE1 (blue rectangle) and transformed into *S. islandicus* LAL14/1 Δarrays cells. The cells are infected with the targeted virus. The homologous recombination (double-crossover) between the virus and the donor DNA generates the desired deletion allele in the viral genome. The crRNA produced from the plasmid-borne mini-CRISPR array is assembled with the endogenous Cas proteins into the CRISPR ribonucleoprotein (crRNP) complex that directs the cleavage of non-recombinant viruses, which are those still harboring the protospacer. Conversely, the recombinant viruses are not targeted and are able to propagate.

The CRISPR-based viral genome editing procedure is depicted in Figure 4. First, two DNA components were cloned into a plasmid. One is a mini-CRISPR array with a spacer targeting the gene to be deleted and the other is a donor DNA with two homologous regions to the viral genome flanking the target gene. Second, the plasmid was transformed into the CRISPR-null host of LAL14/1, Δarrays, and, therefore, the only crRNA is derived from the plasmid-borne mini-CRISPR array. Subsequently, upon viral infection into the transformant, the viral DNA recombined with the donor DNA and the recombinant virus, which has gained a non-target allele, was selectively retained while the input virus was killed due to the presence of a protospacer.

### 3.3. Consecutive Knockout of SIRV2 Accessory Genes

**Figure 5:**
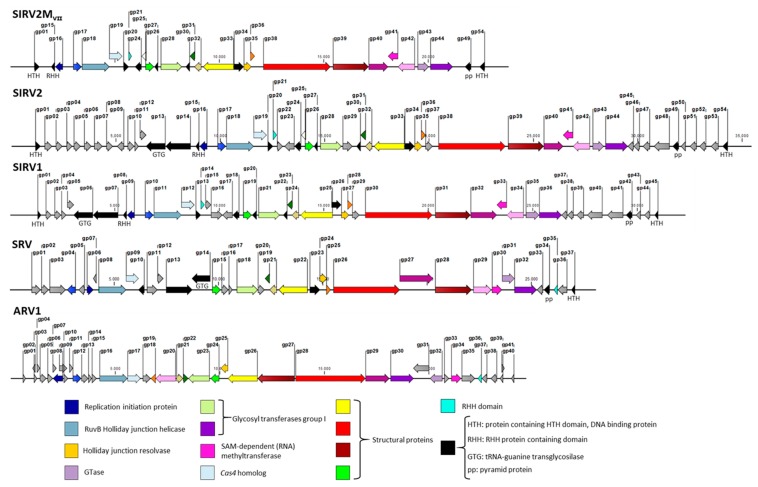
Genome maps of the representative *Rudiviridae* members SIRV2, SIRV1, SRV, ARV1, and the smallest knockout mutant SIRV2M_VII_. Each gene is indicated by a block arrow and their orientation indicates the direction of transcription. Core genes among the *Rudiviridae* family are color-coded while additional genes are shared among SIRVs (in black). Some of these are also present in SRV. SIRV accessory genes are indicated in grey. The white arrow represents *gp25*, which may not be a real gene. Annotations or predicted functions (if available) for genes are indicated.

A total of 23 genes have been identified as accessory genes in the SIRV2 genome on the basis that they do not form part of the core genome of *Sulfolobus islandicus* rod-shaped viruses, which are known as SIRV1-SIRV11 [37]. A 4 kb deletion in the left near-terminus spanning the SIRV2 *gp02* to *gp09* genes was reported recently [29]. The resultant viral mutant, SIRV2M, carries another deletion of a 1.5 kb fragment at the right near-terminus spanning the non-core *gp51* to *gp53* genes, which was revealed by PCR amplification of this genomic region (Table 1). Consequently, after deletion of *gp48* (Bhoobalan-Chitty, in preparation), the viral genome was left with 11 accessory genes: *gp10* (ORF105a), *gp11* (ORF62a), *gp12* (ORF102), *gp22* (ORF91), *gp23* (ORF158b), *gp29* (ORF156), *gp37* (ORF114), *gp45* (ORF94), *gp46* (ORF95), *gp47* (ORF112), and *gp50* (ORF73). Eleven of the SIRVs core genes (black-colored arrows in Figure 5) have no detectable homologs in the genome of the distant member of the *Rudiviridae*, *Acidianus* rod-shaped virus (ARV) and five of these lack detectable homologs in another member known as the *Stygiolobus* rod-shaped virus (SRV). This might be due to sequence divergence and were, therefore, not considered for knockout. In addition, since *gp25* (ORF76) is an ORF that overlaps *gp26* and its expression is extremely low during the entire infection cycle [49,50], it might not be a gene and, therefore, was not considered for knockout (white-colored arrows in Figure 5).

In this part, we explored the CRISPR-based genome editing to knock out the accessory genes one after another. After completion of a gene knockout, the resultant viral deletion mutant is infected into a new host harboring a plasmid for the knockout of the next gene(s). To ensure the purity of the knockout mutants, we diluted virions present in the culture supernatant 10^6^-fold into fresh Δarrays cells carrying the corresponding genome editing plasmid. This process was repeated four to five times. The knockout mutants were named according to the order they were generated from SIRV2M to SIRV2M_VII_ (Table 1).

**Figure 6:**
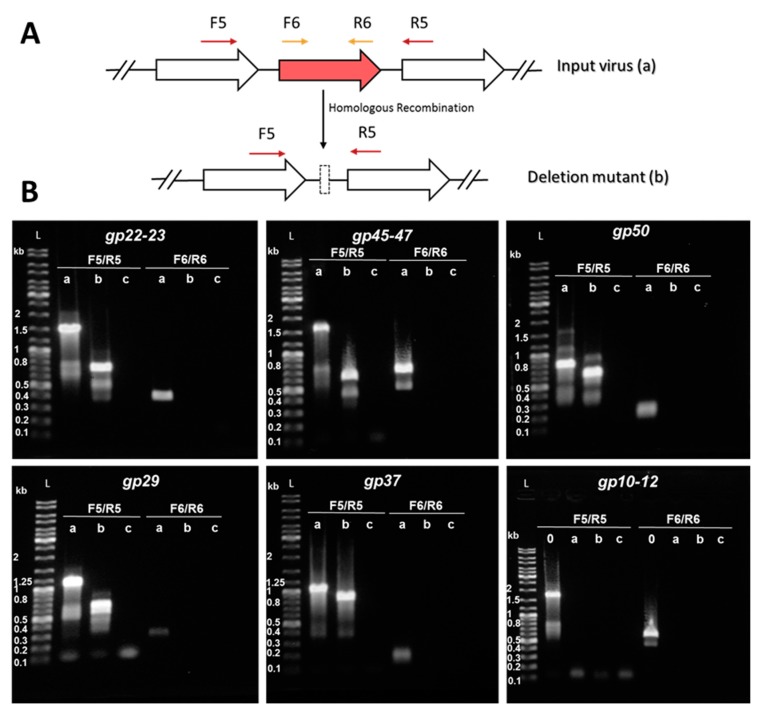
Knockout of accessory genes from SIRV2M_II_ by means of the CRISPR-based genome editing. (**A**) Schematic diagram of the PCR strategy used for screening of mutated gene alleles. F5/R5 primers —indicated by dark red arrows above the genome map—amplify a region covering the deletion target while F6/R6—depicted in gold—amplify a region within the deletion target. Homologous recombination with the donor DNA originates a deletion in the target gene by turning the input virus (a) into a deletion mutant (b). Genes are indicated by block arrows. (**B**) PCR amplification of target gene regions using the supernatant of infected cultures as a template. a, WT-target gene—SIRV2M_II_ as a template— b, deletion mutant allele—SIRV2M_VII_ as a template— c, negative control—water instead of virus template— 0, SIRV2M as a template. The corresponding amplified region is indicated on top of the gel. DNA size ladders (L) with the sizes of selected bands are shown. Primers listed in Table S1 were used.

Potential recombination events and the purity of the knockout mutants were monitored by PCR amplification of the WT-target gene and its deletion mutant allele uses two sets of primers including one annealing upstream and downstream of the target gene (F5/R5) and a second one annealing in the genomic region within the target gene (F6/R6) (Figure 6A). As a result of the recombination between the viral and the donor DNAs, the target gene is expected to be absent from the knockout mutant. Therefore, the amplified fragments by F5/R5 primers would be shorter than that from the input virus while the F6/R6 primer set would not be able to produce any amplicon. Additionally, subsequent DNA sequencing of the PCR fragments allowed the determination of the precise positions of the deletions.

Figure 6B shows the results from PCRs performed using the last deletion mutant (“b” lanes) SIRV2M_VII_ (Table 1) as a template. SIRV2M_II_ which is the starting virus used in this part of the work (Table 1), was used as a positive control (“a” lanes) and water was used as a negative control (“c” lanes). Despite the presence of some minor unspecific bands, all PCR generated major products as expected (Table S2). The genes were knocked out in the following order: *gp22–gp23*, *gp45–gp47*, *gp50*, *gp29*, and *gp37* (Table 1), which were all shown to be present in SIRV2M_II_ and absent in SIRV2M_VII_ (Figure 6B). The three genes, *gp10-gp12*, were supposed to be knocked out in the end, but we discovered, during the knockout trial, that they were already absent from SIRV2M_II_ and were still present in SIRV2M (“0” lane in the last panel of Figure 6B). Moreover, *gp13–gp14,* which are part of the SIRVs core genome but absent in ARV and SRV (Figure 5), were found to be absent in SIRV2M_II_ as well (Table 1). It seems that the deletion of *gp10–gp14* naturally occurred as was the case for *gp02–gp09* and *gp51–gp53* in SIRV2M [29]. The successive propagation of the virus in the CRISPR-null hosts, *S. solfataricus* 5E6 [50] and *S. islandicus* LAL14/1 ∆arrays, could have promoted the loss of these near-terminus genes.

In order to check whether other spontaneous deletions had occurred in the smallest mutant SIRV2M_VII_, five PCR fragments spanning the entire genome were amplified (Figure S1) and sequenced. SIRV2M_II_ was also sequenced as a control. Only a few single point mutations but no other gene deletions were revealed (Table S3). The precise positions of the deletions using the SIRV2 genome as a reference are detailed in Table 1. These were also supported by sequencing of the PCR products derived from each knockout mutant using the respective primers F5/R5.

In all except one case, the knockout yielded the expected deletion. However, the deletion of *gp22–23* was different from what the donor DNA was designed to achieve. In this case, *gp23* was not completely deleted as expected, but, instead, 156 nt at the 5’ end was retained and, unexpectedly, 38 nt at the 3’ end of *gp21* was shown to be lost. Even though *gp21* is a core gene of SIRVs, no obvious homologs are present in ARV or SRV (Figure 5). It is, therefore, difficult to assess its essentiality. Nevertheless, the deletion of the C-terminal 12 aa did not affect the viral life cycle since the knockout mutant can still propagate in the CRISPR-null host (see below).

### 3.4. SIRVs’ Core Genome

After the natural and knockout deletions, the SIRV2 genome has been reduced in a total of 25 genes, which is something that translates into more than a 45% reduction in the gene content. The resultant viral genome, SIRV2M_VII,_ is 23849 kb in length, which is less than 70% of the original genome size. Noteworthy, this points toward the notion that a large portion of viral genomes is comprised merely of accessory genes, which are non-essential for their basic life cycle (grey-colored arrows in Figure 5). Morons are additions [51]—more on them—that may optimize the phage to adapt to a certain host or niche. Changing the combination of these genes gives the virus population access to new niches. 

The remaining genes form the core genome of the 11 members of SIRVs, which are black and color-coded in the genome maps in Figure 5 even though their essentiality remains to be tested by experimental studies. Their average size is substantially larger than that of accessory genes. A comparison between the smallest deletion mutant obtained in this work and representative members of *Rudiviridae* family is depicted in Figure 5. The color-coded genes represent the core genes shared by all the members of the family and it is reflected how they are essential for the lytic cycle of the virus under any condition such as genes encoding the components for DNA replication, nucleotide metabolism, and the assembly of the virus.

### 3.5. Assessing the Effect of The Deletions on Viral Infectivity

**Figure 7:**
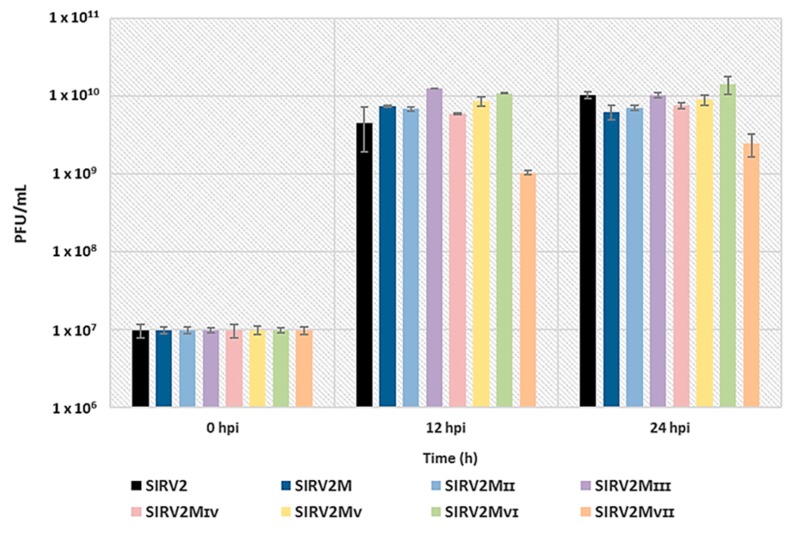
Effect of the deletions on viral infectivity. ∆arrays cells were infected with SIRV2 and all the deletion mutants individually at a MOI of 0.1 and the virus titer (PFU/mL) of the cultures were measured with a plaque assay at 0, 12, and 24 hpi. Results from three technical replicates are shown and error bars indicate the corresponding standard deviation of the mean for the three technical replicates. PFU/mL is plotted in a logarithmic scale.

We compared the infectivity of SIRV2 and all the deletion mutants by infecting the CRISPR-null host, Δarrays, at a MOI of 0.1 and measuring the amount of virions released at 12 h and 24 h post infection (hpi). Figure 7 shows a similar infectivity for SIRV2 and all the deletion mutants except SIRV2M_VII_, which exhibited a tenfold reduction of the viral production. A repeated experiment using SIRV2, SIRV2M_II_, and SIRV2M_VII_ showed a similar result (Figure S2). In addition, the plaques formed by SIRV2M_VII_ were considerably smaller than those from SIRV2M_VI_ and the other tested viruses (Figure S3). Accordingly, it is inferred that the last gene deleted, *gp37*, is not essential but important for the efficient propagation of the virus in the CRISPR-null host.

SIRV2 *gp37* (ORF114) is homologous to proteins encoded by other crenarchaeal viruses including double-stranded DNA genomes, which represent three viral families: *Rudiviridae*, *Lipotrixviridae*, and *Bicaudaviridae* as well as the unclassified *Sulfolobus* turreted icosahedral virus (STIV). Homologs are clearly identified on genomes of SIRV1 (*gp29*, ORF114) [23], SIRV3 (*gp28*, ORF114), *Acidianus* filamentous viruses AFV1 (ORF116) [52] and AFV3 (*gp16*, ORF109) [53], STIV (ORFB116) [54], *Sulfolobus* monocaudavirus SMV4 (*gp61*, ORF113) [55], and *S. islandicus* filamentous virus SIFV (ORF118) [56]. STIV B116 and AFV3-109 have been identified as DNA binding proteins even though the DNA target has not yet been identified [57,58]. Wirth et al. [15] showed that STIV B116 could be knocked out but the resultant mutant exhibited smaller plaques and a delayed infection cycle, which indicates that the null mutant is crippled. Despite DNA binding activity of STIV B116, the knockout mutant showed no change in viral transcription patterns, which suggests that B116 is not a regulator of STIV transcription.

## 4. Conclusions

In this work, *acrID1* was repurposed as a selection marker to genetically modify the SIRV2 genome including knocking out one essential gene. Moreover, using the CRISPR-based genome editing approach, we successfully deleted all the remaining accessory genes of SIRV2, which yields a viral genome formed solely by core genes of the 11 SIRV viruses. Both the Acr-based and the CRISPR-based genome editing approaches were shown to be effective while the former required less work in cloning and the latter lacked markers, which makes consecutive knockout possible. The results provide a good basis for the future functional study of SIRV genes including the identification of new Acrs. This work represents the first example of Acr-based and CRISPR-based viral genome editing in the Archaea domain. The concept is applicable to other virus-host systems in both Bacteria and Archaea.

## Figures and Tables

**Table 1:** SIRV2 and its derivative deletion mutants obtained in previous and present studies. The code number associated to each deletion mutant is indicated. The deletion of the genes is shown in the order at which they were obtained. The precise position of the deletions is indicated by using the SIRV2 genome as a reference. The size of the deletion is also shown.

Virus	Position of New Deletion (bp)	Deletion Size (bp)	Reference
SIRV2	-	-	
SIRV2M: Δ*gp02–09*,*51–53*	Δ1491–5474, Δ32426–33939	3983, 1513	[29]
SIRV2M_II_: Δgp02*–*09,51*–*53,*48*,*10–14*	Δ30803-31479, Δ5678–8557	676, 2879	Bhoobalan-Chitty unpublished
SIRV2M_III_: Δ*gp02–09*,*51–53*,*48*,*10–14*,*22–23*	Δ12664–13395	731	This work
SIRV2M_IV_: Δ*gp02–09*,*51–53*,*48*,*10–14*,*22–23*,*45–47*	Δ29630–30517	888	This work
SIRV2M_V_: Δ*gp02–09*,*51–53*,*48*,*10–14*,*22–23*,*45–47*,*50*	Δ32169–32344	175	This work
SIRV2M_VI_: Δ*gp02–09*,*51–53*,*48*,*10–14*,*22–23*,*45–47*,*50*,*29*	Δ15827–16366	539	This work
SIRV2M_VII_: Δ*gp02–09*,*51–53*,*48*,*10–14*,*22–23*,*45–47*,*50*,*29*,*37*	Δ19910–20119	209	This work

## Supplementary Materials

The following are available online at http://www.mdpi.com/1999-4915/10/12/695/s1, Figure S1: PCR amplification of five fragments spanning the entire viral genomes. Figure S2: Replicate for plaquing efficiency assay assessing the effect of the deletions on viral infectivity. Figure S3: SIRV2MV_II_ exhibits an altered phenotype of small plaques, Table S1: Primer list. Table S2: Expected sizes (bp) from PCR amplification presented in Figure 6. Table S3: Mutations identified in the genome of SIRV2M_II_ and SIRV2M_VII_ after whole genome sequencing using primers listed in Table S1.
