# Supplementary material for: Anti-CRISPR-Based and CRISPR-Based Genome Editing of Sulfolobus islandicus Rod-Shaped Virus 2
Source: Viruses. 2018 Dec 8;10(12):695. doi: 10.3390/v10120695 (PMC6315595; doi:10.3390/v10120695)
Supplement: Supplementary file 1 [file viruses-10-00695-s001.zip › Supplementary figures revised.pdf]

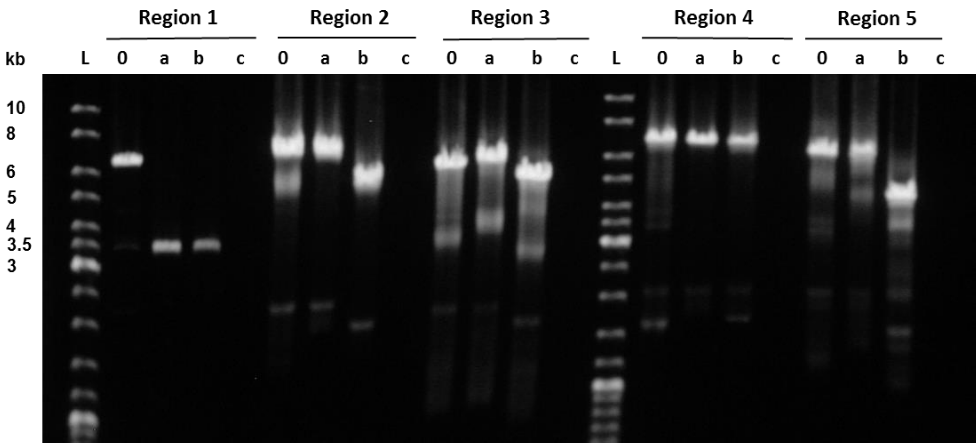

Figure S1. PCR amplification of five fragments spanning the entire viral genomes. The supernatant of infected cultures was used as a template. 0, SIRV2M used as a template; a, SIRV2M$_{II}$ as a template; b, SIRV2M$_{VII}$ as a template; c, negative control, water instead of virus template. The corresponding amplified region is indicated on top of the gel. Primers listed in Table S1 were used. L, DNA size ladder.

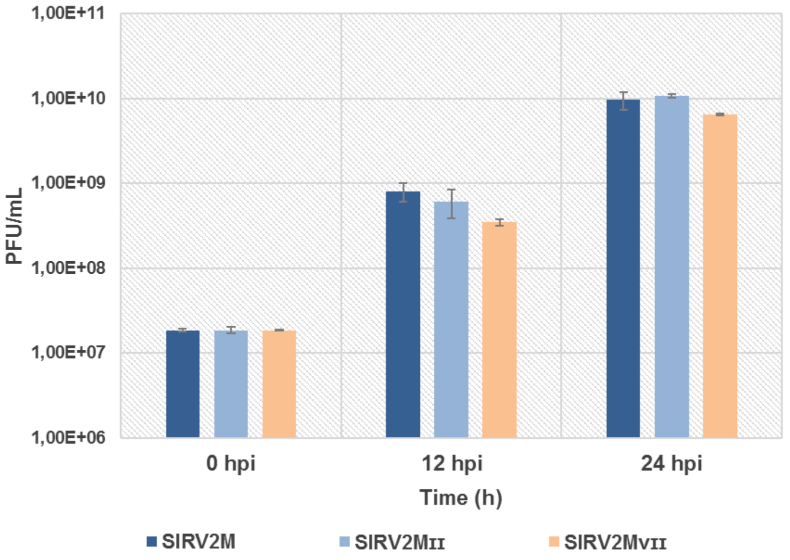

Figure S2. Replicate for plaquing efficiency assay assessing the effect of the deletions on viral infectivity. Δarrays cells were infected with the mutant containing all the deletions (SIRV2M$_{VII}$) and the positive controls SIRV2M and SIRV2M$_{II}$ individually at a MOI of 0.1 and the virus titer (PFU/mL) of the cultures were measured with plaque assay at 0, 12 and 24 hpi. Results from three technical replicates are shown, and error bars indicate corresponding standard deviation of the mean for the three technical replicates. PFU/mL is plotted in logarithmic scale.

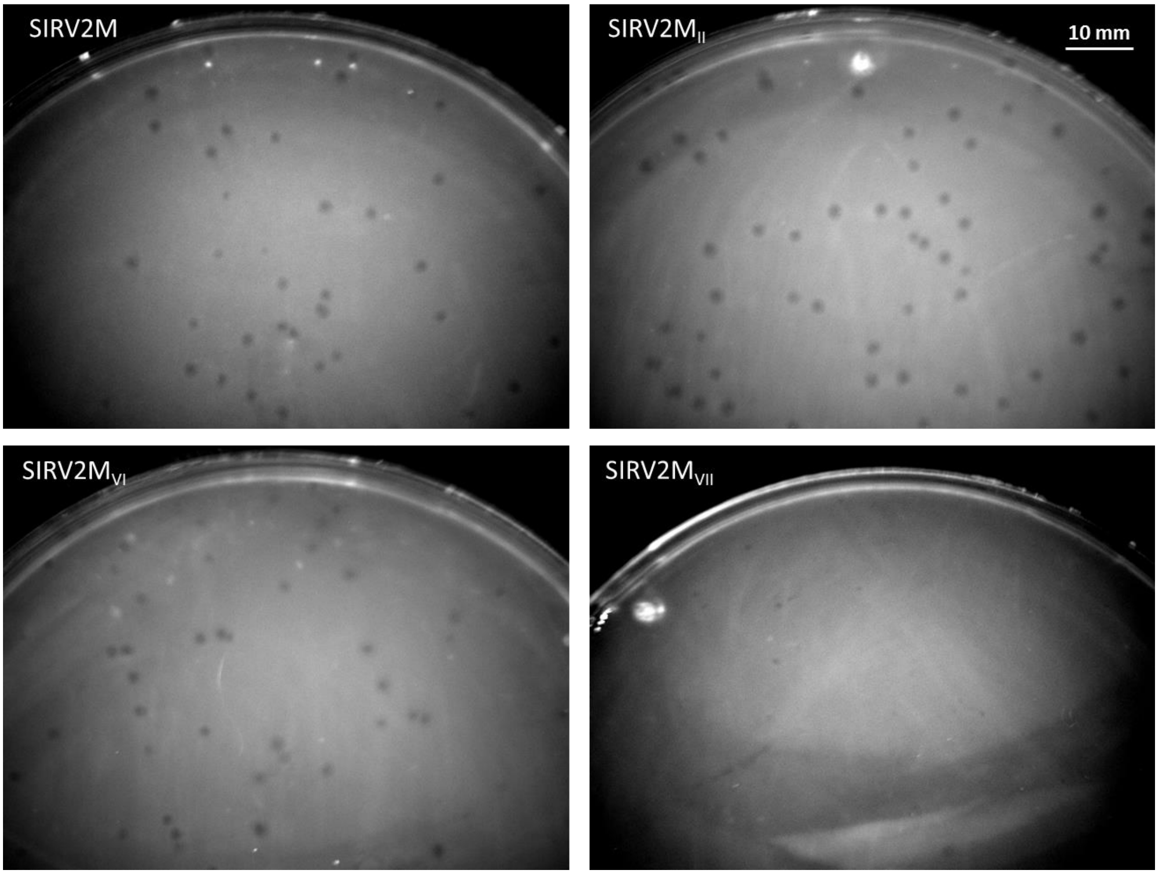

Figure S3. SIRV2M$_{VII}$ exhibits an altered phenotype of small plaques. Plaque size of SIRV2M$_{VII}$ infecting the CRISPR-null host LAL14/1 Δarrays in comparison to those of SIRV2M, SIRV2M$_{II}$ and SIRV2M$_{VI}$.
