# Supplementary material for: Anti-CRISPR-Based and CRISPR-Based Genome Editing of Sulfolobus islandicus Rod-Shaped Virus 2
Source: Viruses. 2018 Dec 8;10(12):695. doi: 10.3390/v10120695 (PMC6315595; doi:10.3390/v10120695)
Supplement: Supplementary file 1 [file viruses-10-00695-s001.zip › Supplementary tables revised.docx]

**Supplementary Table S1. Primer list.**

| **Construct** | **Primer^a^** | **DNA sequence (5’-3’)b** |
| --- | --- | --- |
| pJET1.2/Flankgp29 | *acrID1* *Nhe*IFor | CG*GCTAGC*GTGAAAAAATAAAGAGATAGAGAAGT |
|  | *acrID1* *Kpn*IRev | GG*GGTACC*TAGTTCATAGTTTTAGTTAGTAAGAAG |
|  | gp29Fwd | TTTGTAGCAGAATTTGGATGG |
|  | gp29*Kpn*I*Nhe*IRev | TTATCATGTC*GGTACCGCTAGC*AATCAATAGGACACATATAAAATCC |
|  | gp29*Nhe*I*KpnI*Fwd | CCTATTGATT*GCTAGCGGTACC*GACATGATAAAGGATATGATAAAAGAT |
|  | gp29Rev | CCAAATAAACCAACTGAAGAAG |
| p^gp49^ | promotercsa5*Sph*Ifwd | CAT*GCATGC*TAGAAATGTTTATATAGTGGAATAATAATAGAGAATAAC |
|  | promotercsa5fusionfwd | GTTTATATAGTGGAATAATAATAGAGAATAACATGGCTATAACATTATTAGAAGGAG |
|  | 49*Not*Irev | TT*GCGGCCGC*TTATTTAGCTTGCGTATTTGGAT |
| pJET1.2/Flank49 | *acrID1* *Nhe*IFor | CG*GCTAGC*GTGAAAAAATAAAGAGATAGAGAAGT |
|  | *acrID1* *Kpn*IRev | GG*GGTACC*TAGTTCATAGTTTTAGTTAGTAAGAAG |
|  | 49Fwd | GATACTTTCGATAAATTAAGACTACC |
|  | 49*Kpn*I*Nhe*IRev | TTGACCAGAA*GGTACCGCTAGC*TTTTAATTTTTAAGGTATTATTTTTTTAG |
|  | 49*Nhe*I*KpnI*Fwd | AAAATTAAAA*GCTAGCGGTACC*TTCTGGTCAAAAAATATTATCAAG |
|  | 49Rev | TTTCAGATACTATTTCTAAAGGTTG |
| Recombination check primers | F1 | TCATAATTTCTCATAAACAATTTACTC |
|  | R1 | GATAAAACATTACATATCAAAATTCAT |
|  | F2 | TCATAATTTCTCATAAACAATTTACTC |
|  | R2 | GATAAAACATTACATATCAAAATTCAT |
|  | F3 | GAAGTAGATATCGTATATTCAATTG |
|  | R3 | TTAATACTAAAGAGTTAGCAGATAATG |
|  | F4 | TTATTTAGCTTGCGTATTTGGAT |
| pGE1::gp10-12_Sp/DD | Spacer AAG | AAGTGAAATCTCTTATCTCTAACTTTTAAGATGACTTTCTGA |
|  | Spacer AGC | AGCTCAGAAAGTCATCTTAAAAGTTAGAGATAAGAGATTTCA |
|  | Gp10-12 For Left *PaeI* - F5 | ATTA*GCATGC*AATTTCTGTAACTCATGAGTTAGC |
|  | Gp10-12 Rev Left | TTCATTTAACCAA CATTTTTTCCTCACTATATTTTCGAT |
|  | Gp10-12 For Right | TGAGGAAAAAATG TTGGTTAAATGAAGCTGAAAGACT |
|  | Gp10-12 Rev Right *XhoI* | ATTA*GAGCTC*TTTTACAAATTATAAATGCCTGGATT |
| pGE1::gp22_Sp/DD | Spacer AAG | AAGTATAATTTTTGTGTGGACTTAAATTATTGCATGACATAA |
|  | Spacer AGC | AGCTTATGTCATGCAATAATTTAAGTCCACACAAAAATTATA |
|  | Gp22 For Left *PaeI* - F5 | ATTA*GCATGC* ATAATAGCGTAAGCATAACAG |
|  | Gp22 Rev Left | CTTTTTATTTCATCATTTTTGTAATAATTTCTTTATTCC |
|  | Gp22 For Right | ATTATTACAAAAATGATGAAATAAAAAGTGTAAGC |
|  | Gp22 Rev Right *XhoI* - R5 | ATTA*CTCGAG*GCAAGAAAAGGATTATTAATTC |
| pGE1::gp23_Sp/DD | Spacer AAG | AAGGATTTTAATTTTACTGGAATTGAAGAACCTACTAATTCT |
|  | Spacer AGC | AGCAGAATTAGTAGGTTCTTCAATTCCAGTAAAATTAAAATC |
|  | Gp23 For Left *PaeI* - F5 | ATTA*GCATGC*GGTAGACATTATCGTTTTGAATTG |
|  | Gp23 Rev Left | CTGATAAAATATGTAACTCACTTTCTTTAATCAGAACT |
|  | Gp23 For Right | AGAAAGTGAGTTACATATTTTATCAGTTCTTATTTTCAGT |
|  | Gp23 Rev Right *XhoI* - R5 | ATTA*CTCGAG*GAAATTGGTTAGAAAGTAGATATTC |
| pGE1::gp29_Sp/DD | Spacer AAG | AAGAATGACATATTTAGACCCGTTTTCAAACGATACACAAAC |
|  | Spacer AGC | AGCGTTTGTGTATCGTTTGAAAACGGGTCTAAATATGTCATT |
|  | Gp29 For Left *PaeI* - F5 | ATTA*GCATGC*TTACTAGTTGGCAATGGAATTTAC |
|  | Gp29 Rev Left | GGCTAAAGTAACTTTAGCTACCATTTTTCCACC |
|  | Gp29 For Right | GGTAGCTAAAGTTACTTTAGCCTTTCCTGTTCC |
|  | Gp29 Rev Right *XhoI* - R5 | ATTA*CTCGAG*GAAGAAGAATTAAGAAAAGTATTACC |
| pGE1::gp37_SpI/DD | Spacer AAG | AAGATTCTATTTTTCTCTAATTTTGTATCACATAGTGAATTT |
|  | Spacer AGC | AGCAAATTCACTATGTGATACAAAATTAGAGAAAAATAGAAT |
|  | Gp37 For Left *PaeI* - F5 | ATTA*GCATGC*GTCAGAGCAAAAGAGAAGATC |
|  | Gp37 Rev Left | TCATACAATTTCAGAATTTTATCAATTGTCACTTTTATTGG |
|  | Gp37 For Right | CAATTGATAAAATTCTGAAATTGTATGAAAGTGGAAAAG |
|  | Gp37 Rev Right *XhoI* | ATTA*CTCGAG*CATTACTAAAAAAATACAAAACTAAAAG |
| pGE1::gp45-47_Sp/DD | Spacer AAG | AAGAAGGATCTTTATCTTCAAAAGACAGGTTTGGTCTCTTTA |
|  | Spacer AGC | AGCTAAAGAGACCAAACCTGTCTTTTGAAGATAAAGATCCTT |
|  | Gp45-47 For Left *PaeI* - F5 | ATTA*GCATGC*CGTAGGTAATGGAGTTGAAATG |
|  | Gp45-47 Rev Left | AAATAAGCCTAAGACATCTGATATTTATGAATATAGAGAA |
|  | Gp45-47 For Right | AAATATCAGATGTCTTAGGCTTATTTGCTTTTGTTC |
|  | Gp45-47 Rev Right *XhoI* - R5 | ATTA*CTCGAG*ACCAAGACGGTCAGGAAATGA |
| pGE1::gp50_Sp/DD | Spacer AAG | AAGATAATCTTCTTCATTAATTTCAAAAGAAATCTTAAATTT |
|  | Spacer AGC | AGCAAATTTAAGATTTCTTTTGAAATTAATGAAGAAGATTAT |
|  | Gp50 For Left *PaeI* - F5 | ATTA*GCATGC*CAGCCTTAGCAAAATTCTCATTAT |
|  | Gp50 Rev Left | GGAGAGAAAAATGAGTTAGCAGATAATGAAAAAGATTT |
|  | Gp50 For Right | TCTGCTAACTCATTTTTCTCTCCTCAATATTTTCATAAA |
|  | Gp50 Rev Right *XhoI* - R5 | ATTA*CTCGAG*CAGTTGAAAAGATAGCAAGTTTAC |
| Δgp10-12 check | R5 | TTTGGTGGAATAGTTGCATCTAG |
|  | F6 | AGAGTAGTAGCGAAATTATTGAAG |
|  | R6 | TCCATGAAATCTCTTATCTCTAAC |
| Δgp22 check | F6 | GGTAGATCTATTGATGTTATTTTTA |
|  | R6 | TGTTGTATTATTATGCCAAATCTC |
| Δgp23 check | F6 | TTTTGCTAAAGTTACACCTTTTTGA |
|  | R6 | AGTAGACTTAAAAATTGGAAATGAG |
| Δgp29 check | F6 | AAGAAGTGTGAAAATAGGAAAATGT |
|  | R6 | CTTTATCATGTCTATAATTTTTTCTCT |
| Δgp37 check | R5 | GTCATAAACGTTGGAATATCGG |
|  | F6 | GCCATGATGCTACAATTCAGTT |
|  | R6 | GCTTAATATTTTTCCTTCTTCTAATC |
| Δgp45-47 check | F6 | CTTCAAACTCCATTTTTTTCACC |
|  | R6 | GAGATCTATGCATTCCTTTATATG |
| Δgp50 check | F6 | AAGTGCTAATTCAACTAATTGTCG |
|  | R6 | GAGGAGAGAAAAATAAGTTTTAAAG |
| Positive transformants screening in pGE1/2 | pEXA S | GGCGGTACATAGTGGTACATTAAAG |
|  | pEXA A | GCCCTAACAGATAAGTATAGTAAA |
| Whole genome amplification and sequencing | reg1 For | GTTTTTTTCATTTTTTGCGTAAATTTCG |
|  | reg1 Rev gp18 | CTTAAAATTCTTTCATTATCACGAGG |
|  | reg2 For gp17 | AGAAAAAAAACAGTTGAGGAGGAG |
|  | reg2 Rev gp30 | GAAAAAATTATAGATAGTAAGGAATGG |
|  | reg3 For gp28 | GCTACAGAATTAGAAGATAGAGAAG |
|  | reg3 Rev gp38 | TGGAGTTATCAAGTTATTATATGCGT |
|  | reg4 For gp38 | TACCTAGAAATCCTTTATTTAGACC |
|  | reg4 Rev gp43 | AACTATGTAATCAAAATCTAGTTCATC |
|  | reg5 For gp42 | CTAGCTAAAAGTGAAGAAATTGAAG |
|  | reg5 Rev | AACTAATGAGATAAATTGGAAATTCCA |
| Whole genome sequencing | R1For2 | TTAATTATGTTAGTGTGGAGTATGG |
|  | R1For3 | AATGGAATTTGAAGATTTAGATGTAGT |
|  | R1For4 gp16 | TTTTCAGTATCTCCATATTTATAATTAGT |
|  | R1Rev2 | TTGACGGAAAAGTTTTGGTTTCTC |
|  | R2For2 gp18 | ATGGTTCAGTATTTCTTAGTAATGG |
|  | R2For3 gp19 | AGTGGAAGAATTGATATAGTTTGTG |
|  | R2For4 gp21 | TAGTAGTAGATGAAATGAAAAATGAAC |
|  | R2For5 gp24 | CAGTTCTTATTTTCAGTTTCTGACA |
|  | R2For6 gp26 | TCTCAGCTTTTAACGCAAATCCA |
|  | R2For7 gp28 | GGAAAAATATGGATTTAATGTTACAAG |
|  | R3For2 gp30 | TCTCTATCATATTTCCATAATCTTTCA |
|  | R3For3 gp33 | TGCTTGTCCTTGTAATTGTTCAC |
|  | R3For4 gp33 | TGATTTTGATATTCCGTACTTAACG |
|  | R3For5 gp34 | GAAGAATTACGAAAACTGCTCCA |
|  | R3For6 gp36 | AGAAGATTACAAGTTAAACCAGTTG |
|  | R3For7 gp38 | GATTTAGCTCCAGTTGGATTAGG |
|  | R4For2 gp38 | TGGAATAATAAAAGAATATGAAGATGC |
|  | R4For3 gp38 | ATTTGCCTTCACTTTCAATTATCGA |
|  | R4For4 gp39 | TCATTACAAGACTGGAATAACTTTG |
|  | R4For5 gp39 | CTAATTTATCAAGTGTCTATATTTGGA |
|  | R4For6 gp39 | CCACAATTAACTACAATTTATGTCG |
|  | R4For7 gp40 | TGCAAACAAAATTACGGTCAAATTC |
|  | R5For2 gp44 | AACTATTTTCTATGTCTACCCACAA |
|  | R5For3 gp44 | GTTTAGAAGCATTTATTAGCAAAATCC |
|  | R5For4 gp49 | CATTATATAAGTGGACAATTTCTCC |
|  | R5Rev5 gp47 | TGAAAGAAATGAGTGAACAAAAGCA |
|  | R5Rev4 gp49 | GATCAAAATCCAAATACGCAAGC |
|  | R5Rev3 | GATTTTTTTCATTTTTCAGATACTATTTC |
|  | R5Rev3b gp54 | GAGTCAGTGTAAGAAAAGTTAGG |
|  | R5Rev2 | GAAAAGTTTAAATACCAGAAGAACC |

1. Restriction enzymes used to digest the respective PCR product and the vector (pGE1) before ligation are indicated in the primer names.
2. Restriction sites are indicated in italics and sequences share between primers in fusion PCR are underlined.

**Supplementary Table S2. Expected sizes (bp) of PCR products presented in Figure 6. The region covering the deletion target was amplified using F5/R5 primers; whereas the region within the deletion target was amplified using F6/R6 primers. a, WT-target gene —SIRV2M_II_ as a template—; b, deletion mutant allele —SIRV2M_VII_ as a template—; 0, SIRV2M as a template.**

| **Amplified region** | **Region covering deletion** | | | **Region within deletion** | | |
| --- | --- | --- | --- | --- | --- | --- |
|  | **a** | **b** | **0** | **a** | **b** | **0** |
| **gp22-23** | 1394 | 662 | - | 376 | No band | - |
| **gp45-47** | 1481 | 593 | - | 798 | No band | - |
| **gp50** | 808 | 632 | - | 147 | No band | - |
| **gp29** | 1063 | 614 | - | 292 | No band | - |
| **gp37** | 1047 | 837 | - | 143 | No band | - |
| **gp10-12** | 1606 | 812 | 1606 | 566 | No band | 566 |

**Supplementary Table S3. Mutations identified in the genome of SIRV2M_II_ and SIRV2M_VII_ after whole genome sequencing using primers listed in Table S1.**

| **Mutation** | **Location in SIRV2 (nt)** | | **Affected region** |
| --- | --- | --- | --- |
| C for A substitution | 8732 | Non-coding | |
| A insertion | 14699 | Non-coding | |
| C for A substitution | 14711 | Non-coding | |
| C for T substitution | 16772 | *gp31* | |
| C for T substitution | 20803 | *gp38* | |
| T insertion | 28359 | *gp43* | |
